# Expanding the Rounding Paradigm: Incorporating Inpatient Virtual Rounds Into Infectious Diseases Practice

**DOI:** 10.1093/ofid/ofag249

**Published:** 2026-04-29

**Authors:** Chia-Yu Chiu, Rita Wilson Dib, Takahiro Matsuo

**Affiliations:** Department of Medicine, Division of Infectious Diseases, University of Colorado, Aurora, Colorado, USA; Infectious Diseases Section, Department of Medicine, University of Oklahoma Health Sciences Center, Oklahoma City, Oklahoma, USA; Department of Infectious Diseases, Infection Control, and Employee Health, The University of Texas MD Anderson Cancer Center, Houston, Texas, USA

To  The  Editor—We read with great interest the narrative review by Kiener et al examining rounding styles in inpatient infectious disease (ID) consult services and their implications for education and patient care delivery [1]. The authors provide a thoughtful and practical framework describing traditional rounding approaches, including bedside, walk, table, discovery, hybrid, and double rounding. Their discussion appropriately highlights the inherent tension between efficiency and educational value in consultative environments. However, virtual table rounds could also be considered a form of table rounds as a distinct, increasingly relevant modality.

**Figure 1.**
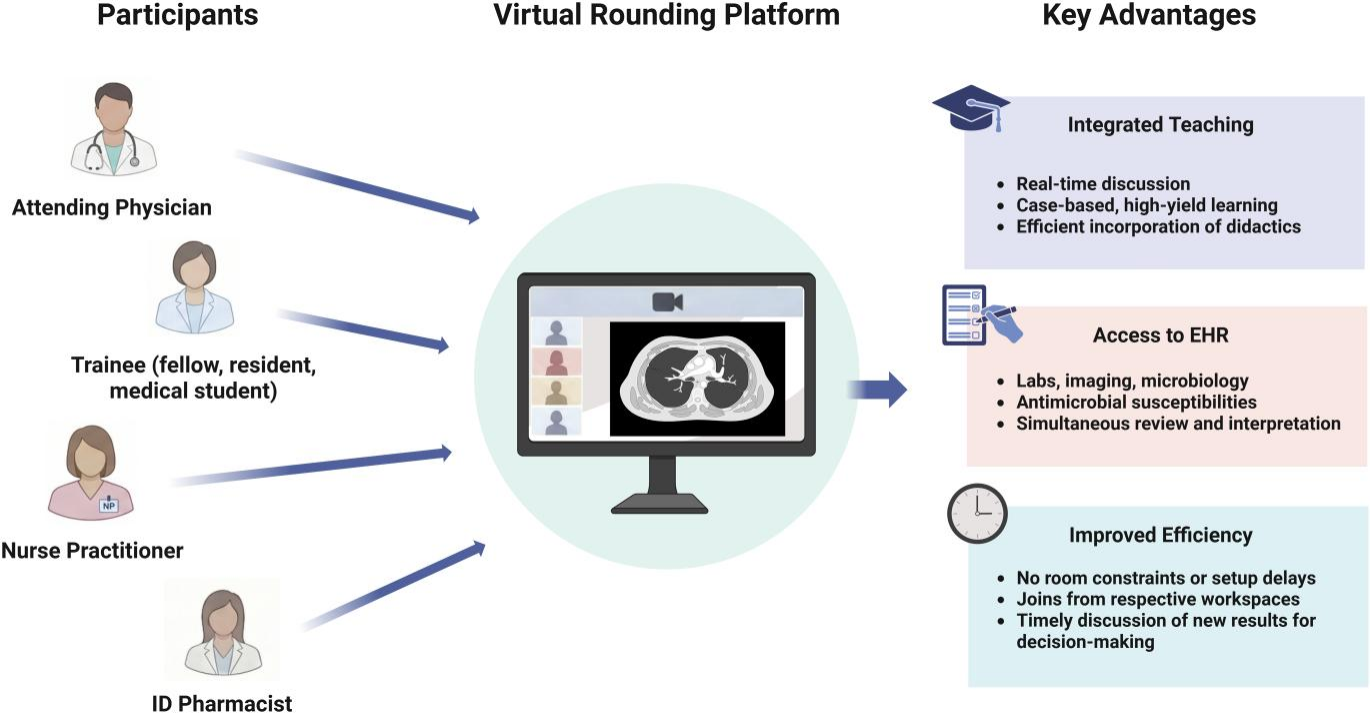
Schema of the virtual table rounds model. Abbreviations: EHR, electronic health record; ID, infectious disease. A virtual platform connects multidisciplinary participants (attending physician, trainees, nurse practitioner, and ID pharmacist) to support integrated teaching, real-time discussion, shared clinical data review via the EHR, and efficient workflow. Created with BioRender.com.

Virtual care models have expanded rapidly since the COVID-19 pandemic, reshaping both clinical care and medical education [2]. While outpatient telemedicine is now well established, with evidence supporting comparable effectiveness in patient outcomes and trainee experience [3, 4], the application of similar principles to inpatient workflows has received far less attention [5–7]. We define inpatient virtual rounds as the use of virtual platforms (eg, Zoom, Microsoft Teams) to conduct structured team discussions involving attending physicians, trainees, nurse practitioners, and ID pharmacists, as a component of table rounds (Figure 1). Drawing on our experience as ID clinicians who have experienced this approach during fellowship and now incorporate it into academic practice, we highlight several educational and operational advantages of virtual rounds.

First, virtual rounds can enhance educational delivery. Virtual platforms allow seamless integration of teaching materials (eg, slides, figures, articles), into real-time discussions, supporting high-yield teaching embedded within clinical care. Similar to in-person table rounds, complex topics can be illustrated visually, effectively transforming case discussions into concise, targeted didactics.

Second, virtual rounds allow simultaneous access to electronic health records enabling all participants to engage with the same information in real time. Rather than relying on handwritten notes or fragmented information displayed on mobile devices, presenters can share complete patient data directly to the audience, including laboratory trends, imaging, antibiotic courses, and susceptibility results. This enables more precise and efficient clinical reasoning. For instance, serial chest imaging can be reviewed collectively to clarify findings suggestive of invasive fungal infection, or pharmacists can simultaneously interpret susceptibility results to guide antimicrobial selection. Compared with traditional walk rounds or discovery rounds, where access to computers may be limited or delayed, this real-time data sharing enhances both decision-making and teaching.

Third, virtual rounds improve workflow efficiency and logistical feasibility. Physical constraints, including limited conference room capacity, competing demands for shared spaces, and delays related to room access and projector setup, frequently hinder in-person table rounding. In addition, geographically dispersed team members can further complicate coordination. In contrast, virtual rounds allow participants to join from their respective workspaces, reducing rounding times, hallway congestion, and facilitating timely discussion of evolving clinical data [6, 7]. This is particularly relevant when new results (eg, antimicrobial susceptibilities, radiology reports) become available during rounds and require immediate integration into management plans.

We acknowledge that the virtual rounds have limitations. To mitigate disengagement, proper rounding form would require participants to turn on their cameras and remain active. However, distractions related to multitasking (eg, replying messages from primary teams, documenting progress notes, or reviewing new consults) are not unique to virtual rounds and are pervasive across all rounding styles in busy inpatient settings. Critically, we do not propose virtual rounds as a complete replacement for bedside rounds. Direct patient interaction remains indispensable for both patient care and trainee bedside skill development. Rather, we view inpatient virtual rounds as a complementary modality, particularly suited for complex case discussions, interdisciplinary collaboration, and data-intensive decision-making. Future discussions of rounding practices in ID consult services should incorporate this modality and further evaluate its impact on education and workflow efficiency from both ID trainee and faculty perspectives, as well as patient outcomes.
